# Multicentric Observational Study on Safety and Tolerability of COVID-19 Vaccines in Patients with Angioedema with C1 Inhibitor Deficiency: Data from Italian Network on Hereditary and Acquired Angioedema (ITACA)

**DOI:** 10.3390/vaccines11040852

**Published:** 2023-04-16

**Authors:** Roberta Parente, Silvio Sartorio, Luisa Brussino, Tiziana De Pasquale, Alessandra Zoli, Stefano Agolini, Ester Di Agosta, Paolina Quattrocchi, Paolo Borrelli, Donatella Bignardi, Angelica Petraroli, Riccardo Senter, Valentina Popescu Janu, Chiara Cogliati, Maria Domenica Guarino, Oliviero Rossi, Davide Firinu, Stefano Pucci, Giuseppe Spadaro, Massimo Triggiani, Mauro Cancian, Andrea Zanichelli

**Affiliations:** 1 Division of Allergy and Clinical Immunology, University of Salerno, 84131 Salerno, Italy; 2 Referral Centre for Systemic Autoimmune Diseases, Fondazione IRCCS Ca’ Granda, Ospedale Maggiore Policlinico di Milano, 20122 Milan, Italy; 3 Allergy and Clinical Immunology Unit, Department of Medical Sciences, University of Torino & Mauriziano Hospital, 10128 Torino, Italy; 4 Allergy Unit, 28100 Novara, Italy; 5 SOD Immunologia Clinica, Azienda Ospedaliera Universitaria Ospedali Riuniti di Ancona, 60126 Ancona, Italy; 6 Immunoallergology Unit, University Hospital of Careggi, 50141 Florence, Italy; 7 Department of Clinical and Experimental Medicine, School and Operative Unit of Allergy and Clinical Immunology, University of Messina, 98125 Messina, Italy; 8 SSD Dermatologia e Allergologia—Ospedale Beauregard, 11100 Aosta, Italy; 9 Department of Medicine Integrated with the Territory, Ospedale Policlinico San Martino, IRCCS Ospedale Policlinico, Genova UO Allergologia, 16142 Genova, Italy; 10 Department of Internal Medicine, Clinical Immunology, Clinical Pathology and Infectious Disease, Azienda Ospedaliera Universitaria Federico II, 80131 Napoli, Italy; 11 Department of Systems Medicine, University Hospital of Padua, 35126 Padua, Italy; 12 Internal Medicine, L Sacco Hospital, ASST Fatebenfratelli-Sacco, 20157 Milan, Italy; 13 Department of Biomedical and Clinical Sciences, University of Milan, 20157 Milan, Italy; 14 Allergy Unit, 62010 Civitanova Marche, Italy; 15 Division of Allergy and Clinical Immunology, University of Cagliari, 09124 Cagliari, Italy; 16 Operative Unit of Medicine, Angioedema Center, IRCCS Policlinico San Donato, San Donato Milanese, 20097 Milan, Italy; 17 Department of Biomedical Sciences for Health, University of Milan, 20097 Milan, Italy

**Keywords:** hereditary angioedema, acquired angioedema, C1 inhibitor, COVID-19, SARS-CoV-2, vaccination

## Abstract

Angioedema due to C1 inhibitor deficiency (AE-C1-INH) is a rare disease characterized by recurrent and unpredictable attacks of angioedema. Multiple trigger factors, including trauma, emotional stress, infectious diseases, and drugs, could elicit angioedema attacks. The aim of this study was to collect data on the safety and tolerability of COVID-19 vaccines in a population of patients affected by AE-C1-INH. Adult patients with AE-C1-INH, followed by Reference Centers belonging to the Italian Network for Hereditary and Acquired Angioedema (ITACA), were enrolled in this study. Patients received nucleoside-modified mRNA vaccines and vaccines with adenovirus vectors. Data on acute attacks developed in the 72 h following COVID-19 vaccinations were collected. The frequency of attacks in the 6 months after the COVID-19 vaccination was compared with the rate of attacks registered in the 6 months before the first vaccination. Between December 2020 and June 2022, 208 patients (118 females) with AE-C1-INH received COVID-19 vaccines. A total of 529 doses of the COVID-19 vaccine were administered, and the majority of patients received mRNA vaccines. Forty-eight attacks of angioedema (9%) occurred within 72 h following COVID-19 vaccinations. About half of the attacks were abdominal. Attacks were successfully treated with on-demand therapy. No hospitalizations were registered. There was no increase in the monthly attack rate following the vaccination. The most common adverse reactions were pain at the site of injection and fever. Our results show that adult patients with angioedema due to C1 inhibitor deficiency can be safely vaccinated against SARS-CoV-2 in a controlled medical setting and should always have available on-demand therapies.

## 1. Introduction

Angioedema due to C1 esterase inhibitor deficiency (AE-C1-INH) is a rare disease characterized by recurrent attacks of angioedema without wheals affecting the skin, abdomen, or upper respiratory system and mediated by bradykinin. This condition can be hereditary or acquired [1]. Hereditary angioedema due to C1 inhibitor deficiency (HAE-C1-INH) is an autosomal dominant disease with an estimated prevalence of 1:50,000 [2] and is caused by mutations in the SERPING1 gene, which codes for C1 esterase inhibitor (a serine protease inhibitor) [3]. There are two types of HAE-C1-INH: HAE type 1 due to C1 inhibitor deficit (85% of HAE patients) and HAE type 2 due to C1 inhibitor dysfunction (15% of HAE patients) [1].

Acquired angioedema due to C1 inhibitor (AAE-C1-INH) is reported with a prevalence ranging between 1:100,000 and 1:500,000. The clinical picture of patients with AAE-C1-INH is similar to that of patients with the hereditary form, but in AAE-C1-INH, the majority of angioedema attacks are located on facial mucosae [4]. AAE-C1-INH often presents in association with B cell lymphoproliferative diseases and/or anti-C1-INH antibodies. Therefore, the pathogenesis of AAE-C1-INH might be explained by the consumption and hypercatabolism of C1-INH [5]. C1-INH deficiency, in both HAE and AAE, causes the dysregulation of the contact system, leading to excessive production of bradykinin with consequent local increasing vascular permeability resulting in angioedema attacks [6]. Angioedema attacks can be caused by emotional factors (anxiety, fear), exercise, drugs, environmental factors, infections, and traumatism. Vaccines can be considered a potential trigger of angioedema attacks, but there are not enough data to confirm this observation. Currently, in Italy, there are six vaccines approved against SARS-CoV-2: two mRNA vaccines (BNT162b2 by BioNTech/Pfizer and mRNA-1273 by Moderna); two viral vector vaccines (ChAdOx1-S by AstraZeneca and Ad26.COV2.S by Johnson & Johnson); one protein subunit vaccine (NVX-CoV2373 by Novavax); one inactivated virus vaccine (VLA2001 by Valneva) [7].

In our country, AE-C1-INH patients were among the first to be vaccinated against SARS-CoV-2 as they were deemed frail because of their complement system disorder, although more recent studies reported no worse outcome than that of the general population for COVID-19. However, COVID-19 was acknowledged as a trigger for angioedema attacks in some patients [8,9,10,11].

Previous studies conducted in relatively small populations of patients with HAE reported that about 10–15% of patients developed angioedema attacks shortly after the injection of COVID-19 vaccines [12,13,14].

Hence, due to the impact of COVID-19 and the vaccine campaign, we performed a multicentric retrospective observational study in order to evaluate the angioedema attack rate and the occurrence of adverse reactions following the vaccination in patients affected by HAE and AAE due to C1 inhibitor deficiency in Italy.

## 2. Materials and Methods

Data from a cohort of 208 patients affected by HAE-C1-INH or AAE-C1-INH were collected. Eleven Italian Center members of the Italian Network on Hereditary and Acquired Angioedema—ITACA (Milan, Florence, Turin, Civitanova Marche, Salerno, Naples, Aosta, Genoa, Ancona, Messina, and Padua) were involved and provided data. Informed consent was obtained from all subjects enrolled in the study. The primary aim of the study is to assess the safety and tolerability of COVID-19 vaccines, evaluating the occurrence of angioedema attacks started within 72 h of the vaccination. We also registered other adverse events reported by patients. The secondary objectives of the study were the evaluation of predictive factors of AE attacks after the vaccine administration and the changes in attack frequency in a six-month period after the vaccination.

### 2.1. Patient Selection

We enrolled in the study 208 adult patients with the diagnosis of HAE-C1-INH or AAE-C1-INH, made according to the World Allergy Organization (WAO)/European Academy of Allergy and Clinical Immunology (EAACI) guidelines [1]. They were on regular follow-ups and underwent the primary vaccination cycle, and some of them underwent the booster vaccination. Patients were vaccinated from December 2020 to June 2022. Patients were interviewed via phone calls or in the outpatient setting during follow-up visits.

### 2.2. Vaccine Use

The administered vaccines were nucleoside-modified mRNA vaccines BNT162b2 (BioNTech/Pfizer) and mRNA-1273 (Moderna) and viral vector vaccines such as ChAdOx1-S (AstraZeneca) and Ad26.COV2.S (Johnson & Johnson). The vaccines were delivered intramuscularly. The dosage for BNT162b2 in the primary vaccination cycle and the booster vaccination was 30 µg/0.3 mL for each dose. As for mRNA-1273, a dosage of 100 µg/0.5 mL in the primary vaccination cycle and a dosage of 50 µg/0.5 mL for the booster vaccination were used, respectively. In regard to ChAdOx1-S and Ad26.COV2.S, respective dosages of 2.5 × 10^8^ Inf.U (infectious units)/0.5 mL and 8.92 log_10_ Inf.U/0.5 mL were used. These dosages were the ones used in the general population.

Due to reports of thromboembolic events linked to viral vector vaccines (Ad26.COV2.S by Johnson & Johnson and ChAdOx1-S by AstraZeneca), in April 2021, the Ministry of Health of Italy recommended these vaccines to people aged 60 or older. Thus, most patients either received nucleoside-modified mRNA vaccines or were administered a viral vector vaccine as the first dose and a nucleoside-modified mRNA vaccine as a second dose and booster dose.

### 2.3. Data Collection

Patient data (age, sex, allergies, type of angioedema, vaccination dates, on-demand therapy, and long-term or short-term prophylaxis possibly started in anticipation of vaccination) were collected for all patients between December 2020 and June 2022.

Data about angioedema attacks and adverse reactions occurring within 72 h of every dose of vaccination were collected. In addition, we collected data on the attack rates per month regarding the six-month period before the first vaccine and the six-month period after the primary vaccination cycle, as well as possible changes in long-term prophylaxis. 

Data on the booster dose were available for 124 patients. We focused on the onset of angioedema attacks and adverse events within 72 h of the administration of the booster dose.

We assessed the severity of attacks, according to their interference with activities of daily living, as: ‘mild’ if no interferences were experienced, ‘moderate’ in case of partial interference, and ‘severe’ for complete incapacity.

### 2.4. Statistical Analysis

Statistics were performed using IBM SPSS Statistics 28.0. The license was granted by the University of Milan. Student’s *t*-test was used for continuous variables, whereas the Chi-square test and, when appropriate, the Fisher test were used for the association of nominal variables. Statistical significance was set at *p* < 0.05.

## 3. Results

### 3.1. Study Population Overview

In total, 208 patients affected by angioedema due to C1 inhibitor deficiency were enrolled. Eleven Italian Centers were involved and provided data about their patients. In our population, 185 patients (89%) were affected by HAE, while 23 patients (11%) were affected by AAE.

The majority of patients were females (118), representing 56.7% of the patient sample. In the subgroups of patients affected by HAE and patients affected by AAE, females made up the majority of the study population, respectively 55.7% (103) and 75.2% (15).

The mean age (±standard deviation, SD) in the total population was 51.4 (±16.9) years. The demographic characteristics of the patients are shown in Table 1. The mean age (±SD) of the population affected by AAE, which was 65.9 (±9.5) years, was higher than that of the population affected by HAE, which was 48.8 (±16.7) years.

We evaluated whether vaccination may have an impact on possible changes in the treatment regimen, mainly on long-term prophylaxis (LTP). We summarized the treatments, both on-demand and LTP, used by patients with HAE and AAE before (Table 2) and after (Table 3) the vaccination. In total, 16 HAE patients changed, started, or stopped a LTP following the primary vaccination cycle, whereas patients with AAE maintained the same treatment regimens after the vaccination.

Twenty-seven percent of patients had a history of allergy (including inhalant allergy, food allergy, and drug allergy).

### 3.2. Angioedema Attacks in Primary Vaccination Cycle

The primary vaccination schedule includes the administration of two vaccine doses for the nucleoside-modified mRNA vaccines and the viral vector vaccine ChAdOx1-S, whereas the viral vector vaccine Ad26.COV2.S only needs one dose. As some patients reported a previous COVID-19 infection, the number of doses administered depended on when the patient was infected by SARS-CoV-2. In our study population, 202 first doses of vaccine were administered (the vaccination executed with Ad26.COV2.S was included in this group). In total, 15 attacks (7.42%) developed within 72 h of the vaccine administration; thirteen of those followed the administration of nucleoside-modified mRNA vaccine. However, BNT162b2 and mRNA-1273 made up 94% of the administered doses (as indicated in Table 4).

As for the second dose of the primary vaccination cycle, 203 doses were administered. In total, 15 angioedema attacks (7.38%) were reported in the 72 h following the vaccination; twelve attacks followed the BNT162b2 vaccination, and three attacks followed the mRNA-1273 vaccination. Even on this occasion, the majority (98%) of vaccines were based on the nucleoside-modified mRNA mechanism (as Table 5 points out). For both the first and second doses of vaccination, no patient affected by AAE was vaccinated with viral vector vaccines.

One hundred twenty-four booster doses were administered, 82 doses were BNT162b2, and 42 doses were mRNA-1273 (respectively 66% and 34% of the administered booster doses, as reported in Table 6). Overall, 18 attacks (14.5%) were reported.

Table 7 shows the characteristics of the 36 patients that reported angioedema attacks, including the site of the attacks and the on-demand therapy used.

**Figure 1:**
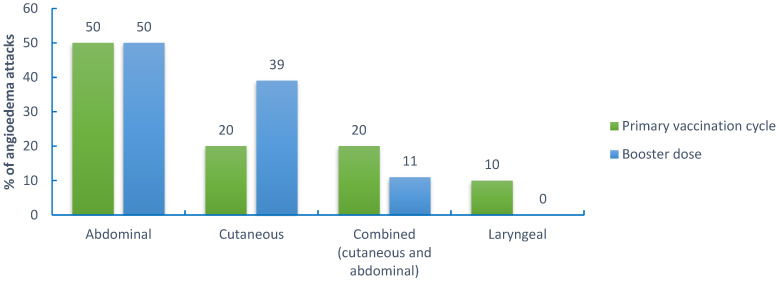
Localization of angioedema attacks following (≤72 h) the primary vaccination cycle and the booster dose.

In the primary vaccination cycle (a total of 405 doses), 50% of attacks involved the bowel mucosa and presented with abdominal cramping. Extremities were involved in 20% of cases, whereas 20% of attacks were characterized by a combined (cutaneous and abdominal) localization. The larynx was involved in only 10% of cases. As for the booster dose (124 doses), abdominal attacks were 50% of total attacks, whereas cutaneous attacks were 39%, and combined (periphery and abdomen) attacks were 11%. No laryngeal attacks were reported after the booster dose (details are shown in Figure 1). In particular, during the whole vaccination schedule, three laryngeal attacks were reported, two attacks related to the first dose, and an attack occurred after the administration of the second dose. However, none of these attacks was severe. The attacks resolved promptly with on-demand therapy or spontaneously in one case, and no hospitalization was required.

**Figure 2:**
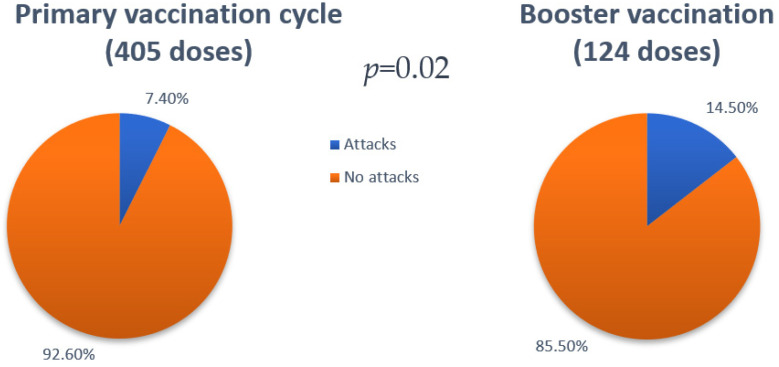
Difference regarding the percentages of doses followed by an angioedema attack (≤72 h) between the primary vaccination cycle and the booster vaccination (7.4% vs. 14.5%—*p* = 0.02).

We noted that the majority of patients (25/36) developed angioedema attacks on one occasion (7 after the administration of the first dose, 7 after the administration of the second dose, and 11 after the booster dose). Only 11 patients (6 females) developed ≥ 2 angioedema attacks across doses. One patient (patient 14 in Table 7) reporting the attack after the first dose underwent premedication with short-term prophylaxis pd-C1-INH before the administration of the following doses and did not report any more attacks. The most used on-demand therapies were plasma-derived C1-INH (in 35.4% of cases) and icatibant (in 37.5% of attacks). Tranexamic acid was used only in one case. A total of thirteen attacks were not treated despite the physician’s recommendation to always treat every attack. Post-vaccination attacks were described as no different from usual attacks and resolved with or without on-demand therapy within 72 h. One patient used both pd-C1-INH and icatibant to treat one attack. Since there was no benefit within 2 h from the administration of pd-C1-INH, the patient self-administered icatibant with attack resolution. Overall, 7.4% of doses administered throughout the primary vaccination cycle were followed by an angioedema attack within 72 h (30 out of 405), while 14.5% of the booster doses were followed by an attack within 72 h (18 out of 124). This difference is statistically significant (*p* = 0.02), as reported in Figure 2. Vaccinations with mRNA-1273 were associated with a higher percentage of attacks post-vaccination in the second dose of the primary vaccination cycle and in the booster dose (respectively 16.7% and 21.4%) in comparison with the attack rate after vaccinations with BNT162b2. Treated attacks resolved within 3 h from the administration of the on-demand therapy, and untreated attacks did not last more than 72 h.

An interesting result is that the percentage of patients on LTP who experienced an angioedema attack after the vaccination is similar to that of patients who are not on LTP (40% vs. 60% for the primary vaccination cycle and 50% vs. 50% for the booster dose). As a matter of fact, the comparison between the two groups shows no statistical significance (*p* > 0.05).

As for allergies, the percentage of attacks after the COVID-19 vaccination was similar between the group of allergic patients and that of non-allergic patients (27% vs. 73%), and there was no statistical difference (*p* > 0.05).

Regarding sex, 17 out of 25 patients who experienced angioedema attacks during the primary vaccination cycle were females, making up 68% of the patients affected by the attacks. Regarding the booster dose, 8 out of 18 patients developing attacks were females, which constituted 44%.

Considering the difference between HAE patients and AAE patients, it can be noticed that two doses for the primary vaccination cycle and one dose for the booster dose in the AAE group were followed by an angioedema attack, and they were related to three different patients. By analyzing the difference between these two groups, though, no statistically significant result was obtained (*p* > 0.05).

### 3.3. Rate of Angioedema Attacks

We collected data about the monthly rate of angioedema attacks in the six-month period before and after the primary vaccination cycle in order to assess a possible variation of this parameter.

**Figure 3:**
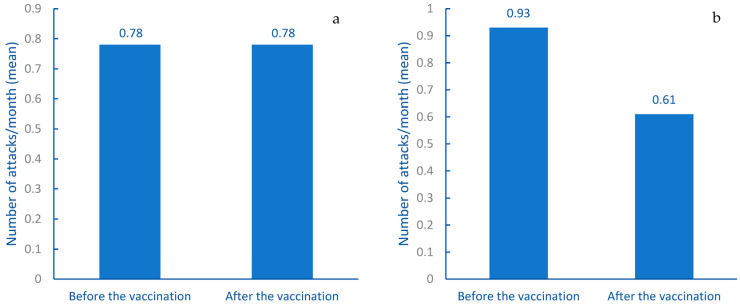
Difference of monthly attack rates between the six-month period before the vaccination and the six-month period after the vaccination in the study population: (**a**) attack rate in patients on on-demand therapy; (**b**) attack rate in patients on LTP.

The results showed a global reduction of the attack rate after the primary vaccination cycle, as the monthly mean attack rate (±standard deviation) before the vaccination was 0.85 (±1.30), while after the vaccination, it was 0.71 (±1.26). This difference was statistically significant (*p* = 0.026). This finding led us to divide the study population into two groups: patients who were on on-demand therapy and patients who were on long-term prophylaxis (including patients who had been on LTP before the primary vaccination cycle and patients who started LTP in the 6-month period following the vaccination). The results are shown in Figure 3. The monthly mean attack rate (±standard deviation) of the former group (114 patients) before the vaccination was 0.78 (±1.20), whereas, after the vaccination, it was 0.78 (±1.46). This result was not statistically significant (*p* > 0.05). As for patients on LTP (94), the monthly mean attack rate (±standard deviation) before the vaccination was 0.93 (±1.42), while after the vaccination, it was 0.61 (±0.96). This difference is of statistical significance (*p* = 0.008). This suggests that the results might have likely been influenced by the use of the LTP. It should be stated that some patients started LTP in the 6-month period before the primary vaccination cycle. Moreover, 16 patients changed, started, or stopped LTP after the vaccination. Thus, the number of patients who actually started LTP after the vaccination was 5, so the number of patients considered in the latter group was 94.

There were no differences in the number of angioedema attacks occurring after the vaccination in relation to the type of disease, gender, or history of previous COVID-19 infection. 

### 3.4. Adverse Reactions

We collected data about adverse reactions that occurred after vaccine administration, focusing on pain and/or erythema at the site of injection, fever, urticaria, dyspnea, and other (myalgia, fatigue). About 33% of first doses were followed by one or more adverse reactions. The types and frequency of adverse reactions related to every type of vaccine are described in Table 8. The 82.4% of patients who reported adverse reactions after the first dose of the vaccine had hereditary angioedema, while 17.6% had acquired angioedema. In addition, 36.8% were males, and 64.2% were females. The majority of adverse reactions occurred after the administration of mRNA-1273 (in 43.8% of cases) or BNT126b2 (in 32.8% of cases). About one-third of doses (36.4%) of ChAdOx1-S was followed by events reported in Table 8. No adverse reaction was reported for Ad26.COV2.S vaccination.

The data about the adverse reactions occurring after the administration of the second dose (38%) are comparable to those collected about the first dose, as indicated in Table 8. As stated above, 89.6% of patients experiencing adverse events were affected by hereditary angioedema, while 10.4% were affected by acquired angioedema. With regards to the percentages of the doses of each type of vaccine followed by adverse reactions, the data were: BNT162b2 35.9%, mRNA-1273 66.7%, and ChAdOx1-S doses did not cause adverse reactions.

In the matter of the booster dose, 65% of the doses caused adverse reactions (Table 8). Considering the characteristics of the patients who reported adverse reactions, 84.6% were affected by hereditary angioedema, and 15.4% were affected by acquired angioedema. Regarding the percentages of the doses of each type of vaccine followed by adverse reactions, the data were: BNT162b2 65.6% and mRNA-1273 64.1%.

Adverse reactions resolved within 72 h from the administration of the vaccines, and patients only used paracetamol, FANS, and/or antihistamines to treat these reactions.

The number of adverse reactions occurring after the booster dose was higher than that of adverse reactions occurring after the doses administered in the primary vaccination cycle. This difference was significant (*p* < 0.0001). The most reported adverse reaction was pain at the injection site, and mRNA-1273 was related to higher percentages of adverse reactions in the primary vaccination cycle than the other vaccines. Furthermore, females reported higher percentages of adverse reactions than males.

## 4. Discussion

The aim of the study was to evaluate the safety and tolerability of SARS-CoV-2 vaccination in a large multicentric cohort of patients affected by angioedema due to C1-INH deficiency. Our patients were administered the standard dosages of the vaccines as used in the general population. We found that 7.4% of vaccine doses exacerbated angioedema attacks in the primary vaccination cycle. If compared to Fijen et al. [12], Ieven et al. [13], and Oztop et al. [14], that respectively reported percentages of 10%, 6.3%, and 8.8% of vaccine doses followed by an attack in HAE patients in the first two doses, our result may be interpreted as comparable, especially considering that most vaccines used in these studies were mRNA based, like in our study. Though, Oztop et al. [14] found a lower incidence of angioedema attacks following the booster dose. It should be stated that in our colleagues’ study [14], only BTN162b2 and DB15806 (Sinovac) vaccines were used and that their study population for the booster dose was almost half our study population. Moreover, our study population included patients affected by AAE who were not included in other studies.

The reported attacks were not more severe than the attacks patients reported before the vaccination, and on-demand therapy was successful in resolving angioedema symptoms. In some cases, the angioedema attacks were not treated by the patients, in contrast with the physician’s recommendation to treat every attack [1,15]. As one of the major concerns about the attacks is the oropharingolaryngeal localization, we reported three cases of attack at this site. All three of them were not severe and did not require hospitalization.

In regard to the booster dose, there was an increase in the percentage of doses followed by an angioedema attack (14.5%) which was statistically significant, suggesting a triggering role for the third immunization.

Another interesting piece of information we gathered was that vaccination with mRNA-1273 was followed by a higher percentage of attacks than BNT162b2. In addition, repeated vaccinations with mRNA-1273 were linked to higher rates of attacks within 72 h of the dose administration in the following doses. In particular, the booster dose with mRNA-1273 was half the dose used in the primary vaccination cycle [7]. However, it should be noted that these results were not significant. We did not notice specific correlations between the occurrence of attacks and vaccination dosages, as standard dosages were used.

Overall, most attacks developed after the administration of nucleoside-modified RNA vaccines. As was previously pointed out by our colleagues [12], mRNA might be able to activate the contact system, promoting the onset of angioedema attacks [16]. Nonetheless, the vast majority of the vaccines used in the study were based on nucleoside-modified RNA technology. Therefore, this may be a relevant contributing factor.

We did not notice significant differences regarding the onset of attacks within 72 h of the administration of the vaccine between patients on on-demand therapy and those on LTP. Consequently, short-term prophylaxis, through the modalities of the guidelines [1], might be an option to consider in case of future vaccinations for patients who developed attacks during previous immunizations. Although, we believe it is best to consider patients who reported an attack after more than one dose. Interestingly, a patient from our study who reported an attack after the first dose and was treated with pd-C1-INH was suggested to use short-term prophylaxis with pd-C1-INH, proving effective in avoiding the occurrence of the attack after the successive doses. As for LTP in our study population, it should be noted that two patients affected by AAE-C1-INH are on LTP with lanadelumab, which is approved only for HAE-C1-INH. Nevertheless, this therapy has proved to be effective in these patients as well, as reported in the literature [17,18]. No significant differences between sex, allergies, and previous infections with SARS-CoV-2 were found.

By comparing the data we gathered and those derived from studies regarding SARS-CoV-2 infection in patients affected by AE-C1-INH, the percentage of patients affected by an attack after the vaccination is lower than that of patients who were infected by the virus, which, according to studies, ranges from 31% to 50% [8,9,10,11].

In terms of attack rates, we can confidently affirm that the primary vaccination cycle did not increase the rate of attacks in our study population. On the contrary, the significant difference that emerged was related to the start of the LTP, which, most likely, caused the reduction of the attack rate in the study population and, in particular, in the group on LTP. It might be interesting to analyze if there are differences regarding the attack rate in the months following the booster dose in patients who underwent the booster vaccination.

As for adverse reactions, the most reported reaction was pain at the site of injection. Overall, in our studies, we collected lower percentages of adverse reactions in the primary cycle than what was found in other studies in the general population [19,20,21]. Similar percentages were reported by Oztop et al. [14] in HAE patients after the primary vaccination cycle. However, for the booster dose, our colleagues found a lower percentage of doses followed by adverse reactions. As we stated earlier, we do not know if this is due to the use of different vaccines (in our study, only mRNA vaccines were used for the booster dose).

Regarding the primary vaccination with BNT162b2, the impact of pain at the site of injection and systemic reactions was similar to that of the literature [19]. Moreover, we also witnessed a higher prevalence in females than in men and in people who were infected by SARS-CoV-2, as already reported by other authors [19]. It must be stated that in the primary vaccination cycle, most of the administered doses were BNT162b2, making the collection of data about this type of vaccine the most consistent. Regarding the primary vaccination cycle with mRNA-1273, most of the adverse reactions were numerically lower in our study than percentages reported in the literature [20], although we also found this type of vaccine to be the one with the highest reactogenicity, in agreement with other authors [22]. In regard to the vaccination with ChAdOx1-S, the administered doses were few. Due to the risk of life-threatening adverse reactions in young people [23], some patients were only administered the first dose and completed the primary vaccination cycle with a nucleoside-modified RNA vaccine. Overall, in both groups who underwent homologous and heterologous immunization with ChAdOx1-S, most adverse reactions were less prevalent than in other studies (except for similar percentages of systemic reactions and pain at the site of injection) [19,21]. It must be noted that for the primary vaccination cycle, the number of doses used of mRNA-1273 and ChAdOx1-S was low; therefore, the collected data about these two types of vaccine must be analyzed with caution when compared to studies with large study populations.

In the matter of the booster dose, slightly more than half of our study population underwent the third immunization. By evaluating the data of both BNT162b2 and mRNA-1273 booster doses, once again, the calculated percentages are lower than those reported in other studies [24,25,26]. Notwithstanding this, the higher proportion of patients who developed adverse reactions resulted in statistical significance if compared to the primary vaccination cycle in our population, implying a relevant role in the onset of adverse reactions for the booster dose. In addition, females reported a higher percentage of adverse reactions than males.

All in all, in our study, the most reported adverse reaction was pain at the site of injection, females were more affected than males, but no difference was noticed between patients affected by HAE and patients affected by AAE. A result that stands out is the percentage of fever reported after every dose. In our population, fever was reported with higher percentages than in other studies [19,20,21,22,24,25,26] for every type of vaccine. Although we are aware of the small size of our population, we do not know if this might be linked to the C1-INH deficiency. For adverse reactions, no specific correlation between the occurrence of particular adverse reactions and vaccination dosages was noticed.

In our population, patients who developed angioedema attacks and/or adverse reactions after vaccination did undergo the following dose(s). Only patients who had SARS-CoV-2 infection before the following dose did not undergo the vaccination. Even for the booster dose, patients did not hesitate to undergo vaccination due to the possible occurrence of angioedema attacks and/or adverse events. Few patients developed an attack following vaccinations more than once.

In consideration of the higher number of angioedema attacks following the booster dose, the decision about undergoing other doses beyond the booster one might be evaluated individually, taking into account various factors involving patients’ health: immunodepression and comorbidities. We hypothesize that repeated vaccinations considered repeated stimuli towards the immune system might consume complement proteins through the stress of a potentiated immunological response, as well as the formation of more immune complexes, which reduce plasma levels of complement proteins and, consequently, of C1-INH.

The main limitation of our study is the small size of the study population, especially if we consider the booster dose. However, angioedema due to C1-INH deficiency is a rare disease; thus, engaging large populations of patients is not easily achievable. Actually, our study, if compared to other studies regarding the SARS-CoV-2 vaccination in patients affected by AE-C1-INH, has recruited a larger cohort. Though, it should be stated that the small amount of data might have contributed to the discrepancy we found between the percentages of adverse reactions we collected and those reported in the literature. Moreover, since this is a retrospective study, another factor to consider is that patients likely remembered systemic reactions more easily than local reactions, as the former has a more relevant impact on everyday life, particularly when conducting a real-life study. As for angioedema itself, it should be reminded that there is a wide variation between patients and in the same patient as well regarding the attack rate over time since it may change throughout life. For instance, patients who are on LTP are usually affected by more severe and frequent angioedema attacks. Lastly, another limitation of our study was the massive employment of nucleoside-modified RNA vaccines, mainly BNT162b2, which restricts the extent of these results.

## 5. Conclusions

In conclusion, vaccination against SARS-CoV-2 in patients affected by angioedema due to C1-INH deficiency is safe, no hospitalization occurred, and no serious adverse reaction was detected.

Furthermore, taking into account the incidence of angioedema attacks in this population affected by COVID-19, vaccination, including the booster dose, is highly recommended as long as performed in a medical setting and patients are provided with on-demand therapy.

## Figures and Tables

**Table 1:** Demographic data of patient population.

	Total	Females	Males
Patients; n (%)	208 (100)	118 (56.7)	90 (43.3)
Age (years); mean (±SD)	51.4 (±16.9)	53.1 (±16.9)	49.5 (±16.9)
Hereditary angioedema; n (%)	185 (100)	103 (55.7)	82 (44.3)
Acquired angioedema; n (%)	23 (100)	15 (75.2)	8 (34.8)

**Table 2:** On-demand therapies and long-term prophylaxes used by the patients before the primary vaccination cycle.

On-Demand Therapy	HAE (*n* = 185)	AAE (*n* = 23)
Icatibant; n (%)	121 (65.4)	15 (65.2)
Plasma-derived C1-INH (Berinert^®^ by CSL Behring); n (%)	93 (50.3)	8 (34.8)
Tranexamic acid; n (%)	9 (4.9)	-
Long-term prophylaxis		
No long-term prophylaxis; n (%)	105 (56.8)	14 (61)
Tranexamic acid; n (%)	2 (1.1)	5 (21.8)
Danazol; n (%)	32 (17.3)	1 (4.3)
Lanadelumab; n (%)	25 (13.5)	2 (8.6)
Experimental ^†^; n (%)	2 (1.1)	-
Plasma-derived C1-INH (Berinert^®^ by CSL Behring); n (%)	6 (3.2)	1 (4.3)
Plasma-derived C1-INH (Cinryze^®^ by Takeda); n (%)	13 (7)	-

^†^ Intended as berotralstat, used for compassionate use.

**Table 3:** On-demand therapies and long-term prophylaxes used by the patients after the primary vaccination cycle.

On-Demand Therapy	HAE (*n* = 185)	AAE (*n* = 23)
Icatibant; n (%)	123 (66.5%)	15 (65.2%)
Plasma-derived C1-INH (Berinert^®^ by CSL Behring); n (%)	91 (49.2%)	8 (34.8%)
Tranexamic acid; n (%)	9 (4.9%)	-
Long-term prophylaxis		
No long-term prophylaxis; n (%)	102 (55.1%)	14 (61%)
Tranexamic acid; n (%)	2 (1.1%)	5 (21.8%)
Danazol; n (%)	30 (16.3%)	1 (4.3%)
Lanadelumab; n (%)	37 (20%)	2 (8.6%)
Experimental ^†^; n (%)	1 (0.5%)	-
Plasma-derived C1-INH (Berinert^®^ by CSL Behring); n (%)	3 (1.6%)	1 (4.3%)
Plasma-derived C1-INH (Cinryze^®^ by Takeda); n (%)	10 (5.4%)	-

^†^ Intended as berotralstat, used for compassionate use.

**Table 4:** No. and type of intramuscularly delivered vaccines as first doses and no. of angioedema attacks following the vaccination (≤72 h).

	Total	BNT162b2	mRNA-1273	ChAdOx1-S	Ad26.COV2.S
No. of vaccination doses (%)	202 (100%)	174 (86.1%)	16 (7.9%)	11 (5.5%)	1 (0.5%)
No. of angioedema attacks (≤72 h) (%)	15 (100%)	12 (80%)	1 (6.7%)	1 (6.7%)	1 (6.7%)

**Table 5:** No. and type of intramuscularly delivered vaccines as second doses and no. of angioedema attacks following the vaccination (≤72 h).

	Total	BNT162b2	mRNA-1273	ChAdOx1-S
No. of vaccination doses (%)	203 (100%)	181 (89.2%)	18 (8.8%)	4 (2%)
No. of angioedema attacks (≤72 h) (%)	15 (100%)	12 (80%)	3 (20%)	0

**Table 6:** No. and type of intramuscularly delivered vaccines as booster doses and no. of angioedema attacks following the vaccination (≤72 h).

	Total	BNT162b2	mRNA-1273
No. of vaccination doses (%)	124 (100%)	82 (66%)	42 (34%)
No. of angioedema attacks (≤72 h)	18 (100%)	9 (50%)	9 (50%)

**Table 7:** Characteristics of the patients who developed an angioedema attack following the vaccination.

	Sex, Angioedema Type	Long-Term Prophylaxis	Dose	Vaccine	Site of Attacks	On-Demand Therapy Used
1	M, HAE	-	First Second Booster	BNT162b2 BNT162b2 mRNA-1273	Combined * Combined Periphery	Icatibant Icatibant Icatibant
2	F, HAE	Cinryze^®^ (by Takeda)	First Second	BNT162b2 BNT162b2	Combined Combined	Pd-C1-INH Pd-C1-INH
3	F, HAE	Berinert^®^ (by CSL Behring)	First Second	BNT162b2 BNT162b2	Abdomen Abdomen	Icatibant Icatibant
4	M, HAE	-	First Second	mRNA-1273 mRNA-1273	Abdomen Abdomen	Pd-C1-INH Pd-C1-INH
5	M, HAE	-	First Second	BNT162b2 BNT162b2	Abdomen Abdomen	Pd-C1-INH Pd-C1-INH
6	F, HAE	-	First	BNT162b2	Periphery	-
7	F, AAE	-	First	BNT162b2	Oropharyngolarynx	-
8	M, HAE	-	First Booster	Ad26.COV2.S BNT162b2	Abdomen Abdomen	Icatibant Icatibant
9	F, HAE	Lanadelumab	First Booster	BNT162b2 BNT162b2	Abdomen Periphery	Pd-C1-INH Pd-C1-INH
10	F, HAE	-	First Booster	BNT162b2 BNT162b2	Abdomen Abdomen	Icatibant Icatibant
11	F, HAE	Lanadelumab	First	BNT162b2	Periphery	Pd-C1-INH
12	F, HAE	-	First	ChAdOx1-S	Abdomen	Pd-C1-INH
13	M, HAE	Lanadelumab	First	BNT162b2	Abdomen	Pd-C1-INH
14	F, HAE	Experimental ^†^	First	BNT162b2	Oropharyngolarynx	Icatibant
15	F, HAE	-	First	BNT162b2	Periphery	-
16	M, HAE	-	Second	BNT162b2	Periphery	-
17	F, AAE	-	Second	BNT162b2	Oropharyngolarynx	Tranexamic acid
18	M, HAE	Lanadelumab	Second Booster	BNT162b2 Bnt162b2	Abdomen Abdomen	Icatibant Icatibant
19	F, HAE	-	Second	BNT162b2	Abdomen	Pd-C1-INH
20	F, HAE	-	Second Booster	BNT162b2 mRNA-1273	Periphery Periphery	- -
21	F, HAE	Berinert^®^ (by CSL Behring)	Second	BNT162b2	Abdomen	Icatibant
22	M, HAE	-	Second	mRNA-1273	Combined	-
23	F, HAE	-	Second	BNT162b2	Periphery	Icatibant
24	F, HAE	Tranexamic acid	Second Booster	mRNA-1273 BNT162b2	Combined Abdomen	Icatibant Icatibant
25	F, HAE	-	Second	BNT162b2	Abdomen	Pd-C1-INH
26	M, HAE	-	Booster	mRNA-1273	Periphery	-
27	M, AAE	Lanadelumab	Booster	BNT162b2	Abdomen	Pd-C1-INH
28	M, HAE	Danazol	Booster	mRNA-1273	Combined	Pd-C1-INH, icatibant
29	F, HAE	-	Booster	BNT162b2	Periphery	-
30	M, HAE	-	Booster	mRNA-1273	Periphery	-
31	M, HAE	Danazol	Booster	mRNA-1273	Combined	-
32	M, HAE	Cinryze^®^ (by Takeda)	Booster	mRNA-1273	Abdomen	Pd-C1-INH
33	F, HAE	Lanadelumab	Booster	BNT162b2	Abdomen	Icatibant
34	F, HAE	Lanadelumab	Booster	mRNA-1273	Abdomen	Pd-C1-INH
35	M, HAE	-	Booster	mRNA-1273	Abdomen	-
36	F, HAE	-	Booster	BNT162b2	Periphery	-

* Combined is attributed to patients experiencing both abdominal and cutaneous attacks. ^†^ Intended as berotralstat, used for compassionate use.

**Table 8:** Percentages of adverse reactions following each vaccination dose.

		Percentage of Adverse Events per Dose	
		Pain at the site of injection	27
		Local erythema	1.1
	BNT162b2	Fever	5.2
		Urticaria	1.7
		Other ^§^	10.3
		Pain at the site of injection	31.3
		Local erythema	5
	mRNA-1273	Fever	18.8
		Urticaria	0
First dose		Other	31.3
		Pain at the site of injection	18.2
		Local erythema	0
	ChAdOx1-S	Fever	36.4
		Urticaria	0
		Other	18.2
		Pain at the site of injection	0
		Local erythema	0
	Ad26.COV2.S	Fever	0
		Urticaria	0
		Other	0
		Pain at the site of injection	26
		Local erythema	0.6
	BNT162b2	Fever	1.1
		Urticaria	6.6
		Other	11
		Pain at the site of injection	44.4
		Local erythema	0
Second dose	mRNA-1273	Fever	33.3
		Urticaria	0
		Other	27.8
		Pain at the site of injection	0
		Local erythema	0
	ChAdOx1-S	Fever	0
		Urticaria	0
		Other	0
		Pain at the site of injection	54.2
		Local erythema	1.7
	BNT162b2	Fever	23.7
		Urticaria	0
Booster dose		Other	22
		Pain at the site of injection	44.1
		Local erythema	5.9
	mRNA-1273	Fever	26.5
		Urticaria	2.9
		Other	41.2

^§^ Systemic reactions not included in the other categories (myalgia, fatigue).

## Data Availability

The data presented in this study are available on request from the corresponding author. The data are not publicly available due to privacy and ethical restrictions.
